# Implementing structured functional assessments in general practice for persons with long-term sick leave: a cluster randomised controlled trial

**DOI:** 10.1186/1471-2296-10-31

**Published:** 2009-05-06

**Authors:** Nina Østerås, Pål Gulbrandsen, Jūratė Šaltytė Benth, Dag Hofoss, Søren Brage

**Affiliations:** 1 Section of Occupational Health and Social Insurance Medicine, Institute of General Practice and Community Health, Faculty of Medicine, University of Oslo, Oslo, Norway; 2 HØKH, Research Centre, Akershus University Hospital, Akershus, Norway; 3 Faculty Division Akershus University Hospital, University of Oslo, Oslo, Norway

## Abstract

**Background:**

The increasing attention on functional assessments in medical and vocational rehabilitation requires a focus change for the general practitioners (GP) into paying attention to patient resources, possibilities and coping instead of symptoms, problems and limitations. The GPs report difficulties in performing the requested explicit functional assessments. The purpose of this study was to implement a structured method in general practice for assessing functional ability in persons with long-term sick leave. The study aim was to evaluate intervention effects on important GP parameters; knowledge, attitudes, self-efficacy towards functional assessments and knowledge about patient work factors.

**Methods:**

Fifty-seven GPs were randomly assigned to an intervention or a control group. The intervention group GPs attended an introductory one-day work-shop and implemented structured functional assessments during an eight months intervention period. GP knowledge, GP attitudes, and GP self-efficacy towards functional assessments, as well as GP knowledge of patient work factors, were collected before, after and six months after the intervention period started. Evaluation score-sheets were filled in by both the intervention GPs and their patients immediately after the consultation to evaluate the GPs' knowledge of patient work factors.

**Results:**

The intervention GPs reported increased knowledge (B: 0.56, 95% CI (0.19, 0.91)) and self-efficacy (B: 0.90, 95% CI (0.53, 1.26)) towards functional assessments, and increased knowledge about their patients' workplace (B: 0.75, 95% CI (0.35, 1.15)) and perceived stressors (B: 0.55, 95% CI (0.23, 0.88)) with lasting effects at the second follow-up. No intervention effect was seen in relation to GP attitudes. Both before and after the intervention, the GPs were most informed about physical stressors, and less about mental and work organisational stressors (Guttman's reproducibility coefficient: 0.95 and 1.00). After the consultation, both the intervention GPs and their patients reported that the GPs' knowledge about patient work factors had increased (GP B: 0.60 (95% CI: 0.42, 0.78); patient B: 0.50 (95% CI: 0.34, 0.66)).

**Conclusion:**

Introducing and implementing structured functional assessments in general practice made the GPs capable to assess functional ability of their patients in a structured manner. Intervention effects of increased GP knowledge and GP self-efficacy sustained at the second follow-up.

## Background

Assessments of patients' functional ability are necessary in medical and vocational rehabilitation. To an increasing extent, general practitioners (GP) in the European countries are being asked to assess function, in addition to disease and illness, in social security claims [1,2]. This focus on functional ability is unfamiliar to GPs [3]. It represents a shift in their attention from patient symptoms, problems and limitations into resources, possibilities and coping. Earlier, functional assessments have been an implicit part of their practice, whereas at present an explicit communication of functional abilities is required. The GPs reported difficulties and were reluctant to meet this request [3]. This was due to lack of training and guidelines, as well as confusing terminology and insufficient knowledge of specific occupational demands [3]. In 2003 only 35% of the GPs in Norway met the request for functional assessments in sickness certification forms [4]. Additionally, the GPs' procedures for functional assessments are usually non-standardised and strongly influenced by their personal and professional interest in functional assessments and working life in general [3].

Methods for structured functional assessment have been developed and tested in some countries, including England and Finland [5-7], but to our knowledge there is no previous randomised controlled study directed at functional assessments of persons with long-term sick leave in general practice. The many randomised, controlled studies addressing professional educational or quality assurance interventions carried out to improve quality of care, show that active multifaceted approaches are more likely to be effective compared to passive single interventions [8].

Based on these experiences, a structured method for functional assessments of persons with long-term sick leave in general practice was developed and tested by GPs in a cluster randomised controlled trial. The purpose was to provide a tailor-made, structured functional assessment method for GPs in busy and ordinary primary care practices. The method was designed to be appropriate for assessing and communicating functional ability information along with suggestions for workplace adjustments to local social security officers and employers. Intervention effects on patient sick leave will be reported elsewhere.

### Study objectives

The first aim of this study was to assess intervention effects on GP knowledge, GP attitudes and GP self-efficacy towards functional assessments. The second aim was to assess intervention effects on GP knowledge about their patients' perceived physical, mental and organisational stressors at the workplace. The third aim related to the patient level and was to assess whether the intervention GPs and their patients had similar evaluations of the GPs' knowledge about patient work factors immediately after the consultation.

## Methods

### Study setting and sample

With the assistance of the Section of General Practice, University of Oslo, and of local medical consultants, 360 GPs in the south-eastern part of Norway were identified and written invitations were sent in November 2004. The responders were randomly assigned to the intervention or the control group according to a computer generated randomisation list made by an independent researcher. The researchers were not blinded to group allocation.

The intervention GPs were requested to apply the intervention on ten consecutive sick-listed persons. The criteria for including a sick-listed person were: being part-time or full-time sick-listed for between eight and 26 weeks and having good prospects of a return to work, meaning that the GPs should exclude persons they thought were candidates for permanent disability benefits.

Informed written consents were received from all GPs. For reasons of anonymity, no written consent was collected from their patients, but the GPs asked their patients for a verbal informed consent. The Regional Committee for Medical Research Ethics and The Norwegian Data Inspectorate approved the study.

### Sample size

Using a table for sample size determination [9] we specified a power of 80% to detect a medium-sized difference of 1.2 standardised effect size in relation to knowledge about functional assessments at the GP level with a significance level of 5%. We found the required sample size to be 22 GPs in each group.

### The intervention

The target for the multifaceted intervention was the intervention group GPs. The structured functional assessment method was introduced at a one-day workshop including teamwork and role-playing. The need to practice the assessments as part of the process of the trial was acknowledged, and the project group provided phone support when needed. The workshop was accredited by the Norwegian Medical Association for continuing medical education points. The intervention GPs were requested to apply the intervention method on their patients according to the inclusion and exclusion criteria mentioned above.

The included patients were asked to self-report their functional abilities prior to the GP-consultation using the Norwegian Function Assessment Scale (see Additional file 1). This instrument was developed by an expert group in social insurance in 2000 to assess the need for rehabilitation, adjustment of work demands among sick-listed persons as well as the rights to social security benefits [10]. It comprises 39 items derived from the activities/participation component in the International Classification for Functioning, Disabilities and Health [11]. The items are relevant for assessing physical and mental functioning in working life, some relating to activities of daily living [12,13]. The sick-listed persons were also asked to self-report work exposures and perceived stressors at work prior to the GP-consultation using the Work Description Form (see Additional file 2).

During the consultation, the GP independently assessed the patient's functional abilities on the basis of the two forms, the patient's medical history, clinical findings, and motivation. The assessment was formalised as the Function Assessment Report (see Additional file 3), which was sent to the employer and the local social security office. This whole procedure was expected to take about 40 minutes. The GPs in the control group were requested to assess functional ability as usual during the intervention period: March – October, 2005.

### Outcome measures

#### GP knowledge, attitude, self-efficacy and GP knowledge of patient perceived stressors

The quality of the educational and implementation components of the intervention was measured in the main questionnaire, which was tailor-made for this study by the project group. The main questionnaire included 19 items (see Additional file 4), and the first item mapped self-reported GP knowledge about functional assessments. Four items were constructed to cover GP attitudes towards functional assessments (items no. 4 and 7–10), and three items were made to assess GP self-efficacy towards performing such functional assessments (items no. 11, 13 and 14). Of the remaining 11 items, five were related to GP knowledge about patient work factors: the workplace (item no. 15), the work tasks (item no. 16), and the perceived stressors at work (items no. 17–19). The last five items were included for other purposes and not relevant in this study (items no. 2, 3, 5, 6, and 12). Knowledge and attitude were assessed since they represent the first and second process stage for adopting new ideas or changing behaviour [14]. Self-efficacy (mastering beliefs) was measured since it might influence the initial decision to perform behaviours, the effort expended, and the length of time the individual will persist in the face of obstacles and aversive experiences [15]. All items in the main questionnaire were scored along a five all-point defined scale from very poor to very good, or from totally disagree to totally agree. There was an inverse scoring for one item (no. 10). A small group of GPs pilot tested the questionnaire beforehand.

The main questionnaire was completed by the randomised GPs at three time points: immediately before (T0) and after the intervention period (T1), and at the follow-up six months later (T2). Two written and one oral reminders were given to non-respondents.

#### GP and patient evaluations

The evaluation score-sheet was used to measure the performance of the functional assessment method itself and how it influenced the GPs' knowledge about patient work factors (see Additional files 5 and 6). It was filled in by both the GP and the patient immediately after the GP-consultation. The GP and the patient rated the GP's knowledge level on two items using a five all-point defined scale from no knowledge to exceptionally good knowledge. The first and the second item were related to the GP knowledge level before and after the consultation, respectively. The patients' evaluations can be seen as a validation of the GPs' evaluations.

#### Descriptive data

Information on characteristics of the GPs was collected to allow comparisons with national data: gender, age, speciality in family medicine, working hours per week, number of consultations per day, and list size. For reasons of anonymity, only the consultation date, gender and age for the included patients were registered.

### Statistical analyses

**Figure 1:**
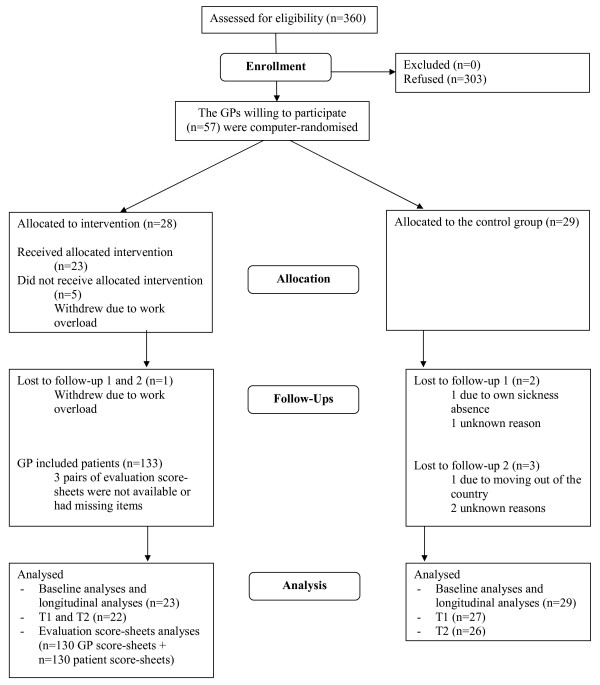
**Flow chart of participants through trial**.

The scores from all participating GPs were included in the baseline and the longitudinal analyses, although three, and then four GPs did not return the two follow-up questionnaires, respectively (Figure 1). Two methods of imputing missing values, last-observation-carried-forward (LOCF) and the median imputation, were applied for single items missing and for total questionnaire missing at T1 and T2 (those lost to the follow-ups). This did not change the conclusions, so results from the original non-imputed dataset are presented. Non-parametric and parametric tests for independent samples were used to compare subgroups and to compare participants' descriptive data with national data.

#### GP knowledge, attitude and self-efficacy towards functional assessments

Confirmatory factor analyses using AMOS [16] were used to test the main questionnaire data against hypothesised model structures. As a result, 11 of the included 14 items sum to form three domains (χ^2 ^(df = 59) = 71.645, p = 0.125): GP attitudes (items no. 4 and 7–10), GP self-efficacy (items no. 11, 13 and 14) and GP knowledge about patient perceived stressors at work (items no. 17–19). Domain scores for these three domains were calculated by adding the item scores and dividing by the number of items completed. The remaining three items (no. 1, 15 and 16) were used as single items in the following analyses.

Non-parametric tests for two related samples were used to analyse domain and item score changes in attitude, self-efficacy and knowledge between two time points, whereas the linear mixed model for repeated measurements using SPSS (version 14.0.2) was estimated to assess longitudinal score changes. This linear mixed bi-level model was chosen for the longitudinal analyses because it allows missing item values. All variables were treated as fixed effects including the intercept and an interaction variable: time by group. The three domain scores and the three single item scores were used as dependent variables. The covariance (among repeated measures of dependent variables on the same individuals) model was chosen using Akaike's Information Criterion [17], and compound symmetry structure was the best. With compound symmetry covariance it is assumed that the variance is constant across occasions [18], and a close examination of dependent variable correlations between different time points showed low variations. The intervention effect was assessed by the interaction term to analyse if the scores of the two groups differed in time from T0 to T1 (the type III Wald tests, p < 0.05). To analyse the stability of the intervention effect at the second follow-up, T2 was compared with T1. All estimates in the multivariate models were adjusted for GP gender, age and number of daily GP consultations.

#### GP knowledge of patient perceived stressors

To assess the potential grading of GP knowledge about their patients' perceived physical, mental and work organisational stressors at the workplace at T0 and T1, the Guttman's reproducibility coefficient [19] was calculated. Guttman's reproducibility coefficient shows which fraction of the responses to a set of questions designed to measure one dimension that fits the cumulative pattern. It can be read as the chance to predict correctly the responder's answer to any given question on the basis of his/her sum-score (i.e., sum of endorsed items in a set of questions). A Guttman's reproducibility coefficient of 1 means that all responders with a sum-score of 1 achieved their one point on the "easiest" question to agree to, all those who scored 2 points got their points by agreeing to the two "easiest" questions etc. To conclude that the observed data fit a Guttman-scale, the reproducibility coefficient should exceed 0.90 [20].

#### GP and patient evaluations

Evaluation score-sheet data was analysed by two separate linear mixed models for repeated measurements with patients (level 1) nested within the intervention group GPs (level 2). All variables were treated as fixed effects including the intercept, and compound symmetry structure was used as covariance model because before and after scores were correlated. The dependent variables were the GP-evaluated and the patient-evaluated knowledge scores after the consultation. Estimates were adjusted for the GP-evaluated and patient-evaluated knowledge scores before the consultation, GP and patient gender and age as well as the number of daily GP consultations.

## Results

### Sample characteristics

Of the 360 GPs invited, 57 (15.8%) agreed to participate (Figure 1). No information was obtained about non-respondents. Missing item values were few, ranging 0.0 – 1.9% for the main questionnaire and 0.8% for the two evaluation score-sheet items. The GPs in the intervention group applied the intervention on a total of 133 sick-listed persons (2–10 per GP). For these patients, the mean age was 44.8 years and the percentage of males was 31.5%. The mean age and the percentage of males among long-term sick-listed persons on a national basis for the same period, was 42.0 years and 37.5% respectively [21]. A small proportion of the intervention GPs needed some phone support and guidance in the beginning.

There was no significant difference between the intervention group and the control group at T0 with respect to background information (Table 1). Compared to all GPs in Norway, the proportion of female GPs and the GPs' mean age in the study sample were slightly higher, but the difference was not significant. The proportion of specialists in family medicine and the list size for the participating GPs were significantly higher than the corresponding national numbers (p < 0.05)[22].

**Table 1:** Sample characteristics of the participating GPs and corresponding national data for general practice GPs, 2005.

	**Intervention group**	**Control group**	**Norwegian general practice GPs**^1^
	(n = 23)	(n = 29)	(n = 3757)
**Females, n (%)**	8 (34.8)	11 (38.0)	1145 (30.5)
**Males, n (%)**	15 (65.2)	18 (62.1)	2612 (69.5)
			
**Speciality in Family Medicine, n (%)**	16 (69.6)	24 (82.8)	2217 (59.0)*
			
**Mean age, y (SD)**	49.3 (10.4)	49.5 (8.7)	47.9
**Mean Weekly working hours, h (SD)**	37.5 (7.2)	41.3 (8.5)	-
**Mean daily consultations, n (SD)**	21.8 (4.7)	21.0 (4.8)	-
**Mean list size, n (SD)**	1254.1 (397.4)	1309.8 (210.0)	1189.0*

^1 ^Numbers from The Norwegian Labour and Welfare Administration [22]

* p < 0.05

### GP knowledge, attitude and self-efficacy towards functional assessments

No significant difference between the two groups was found at baseline in relation to GP knowledge, GP attitudes, or GP self-efficacy. At T1 the intervention group reported significantly (p < 0.05) more knowledge about functional assessments, the patients' workplace, work tasks, and perceived stressors as well as higher self-efficacy regarding functional assessments (Table 2). The change in mean scores ranged 0.5 – 0.8 for the five-point scale. There were ignorable or no changes in mean GP attitude scores. In the control group there were no changes in the scores. The stability of the mean scores was tested at T2, and no significant changes in mean scores were seen in the intervention group, whereas in the control group the mean score for knowledge about perceived stressors increased significantly.

**Table 2:** Cluster level analyses (GP level) on knowledge, attitudes and self-efficacy in intervention and control groups.

		**T0**	**T1**	**T2**
		**n = 23^a ^+ 29^b^**	**n = 22^a ^+ 27^b^**	**n = 22^a ^+ 26^b^**
		**Mean (95% CI)**	**Mean (95% CI)**	**Mean (95% CI)**
GP knowledge about functional assessments	**IG**	**3.1 (3.0, 3.2)**	3.8 (3.5, 4.1)*	**3.8 (3.5, 4.1)**
	CG	3.2 (3.0, 3.4)	3.3 (3.1, 3.6)	3.4 (3.1, 3.7)
				
GP attitude towards functional assessments	**IG**	**4.0 (3.7, 4.3)**	**4.1 (3.8, 4.4)**	**4.0 (3.7, 4.3)**
	CG	4.1 (3.9, 4.3)	4.1 (3.8, 4.4)	3.8 (3.5, 4.1)
				
GP self-efficacy	**IG**	**3.1 (2.8, 3.4)**	3.9 (3.6, 4.2)**	**3.8 (3.5, 4.1)**
	CG	3.5 (3.3, 3.7)	3.4 (3.1, 3.7)	3.5 (3.2, 3.8)
				
GP knowledge about the workplace	**IG**	**2.8 (2.5, 3.1)**	3.6 (3.3, 3.9)*	**3.6 (3.3, 3.9)**
	CG	2.9 (2.6, 3.2)	2.9 (2.6, 3.2)	3.1 (2.8, 3.4)
				
GP knowledge about the work tasks	**IG**	**3.1 (2.8, 3.4)**	3.6 (3.3, 3.9)*	**3.6 (3.4, 3.8)**
	CG	3.1 (2.9, 3.3)	3.1 (2.9, 3.3)	3.4 (3.1, 3.7)
				
GP knowledge about perceived stressors	**IG**	**3.0 (2.8, 3.2)**	3.5 (3.3, 3.7)*	**3.3 (3.0, 3.6)**
	CG	2.9 (2.7, 3.1)	2.9 (2.7, 3.1)	3.1 (2.9, 3.3)*

Mean scores with 95% confidence intervals at three time points.

* p < 0.05, ** p < 0.01

The p-values are based on non-parametric tests for related samples between T0 and T1, and T1 and T2.

^**a**^: Intervention group, ^**b**^: Control group

T0: Immediately before the intervention period started, T1: Immediately after the intervention period ended, T2: Six months after the intervention period ended

IG: Intervention group, CG: Control group

Both crude and adjusted longitudinal analyses were done, but since estimates and standard errors were very similar, only the adjusted estimates are shown in Table 3. A significant (p < 0.05) intervention effect was found for GP knowledge about functional assessments, GP self-efficacy, and GP knowledge about the patients' workplace, work tasks, and perceived stressors, but not for GP attitudes. Adjusted estimates ranged 0.6–0.9 for the five-point scale. Increasing GP age was significantly associated with increasing knowledge about the patients' workplace and perceived stressors. The intervention effect remained evident at the second follow-up, except for knowledge about perceived stressors. Seven of the intervention GPs stated at the second follow-up that they had continued to use the structured functional assessment method, although they were no longer equivalently paid for prolonged consultations.

**Table 3:** Cluster level (GP level; n = 52) longitudinal analyses on knowledge, attitudes and self-efficacy.

	**Multivariate (adjusted)**			
**Dependent and independent variables**	**Estimate**	**SE**	**p-value**	**95% CI**
**GP knowledge about functional assessments**				
Time × group (T0 – T1)	0.56	0.18	0.003	0.19, 0.91
Time × group (T1 – T2)	-0.12	0.18	0.500	-0.48, 0.24
GP gender	-0.05	0.15	0.757	-0.35, 0.26
GP age	0.01	0.01	0.148	-0.01, 0.03
No. of daily consultations	0.02	0.02	0.126	-0.01, 0.06
				
**GP attitude towards functional assessments**				
Time × group (T0 – T1)	0.20	0.16	0.231	-0.13, 0.52
Time × group (T1 – T2)	0.13	0.16	0.420	-0.19, 0.46
GP gender	0.17	0.18	0.361	-0.20, 0.54
GP age	-0.00	0.01	0.741	-0.02, 0.02
No. of daily consultations	0.02	0.02	0.402	-0.02, 0.06
				
**GP self-efficacy**				
Time × group (T0 – T1)	0.90	0.18	<0.001	0.53, 1.26
Time × group (T1 – T2)	-0.13	0.19	0.478	-0.50, 0.24
GP gender	-0.28	0.19	0.143	-0.66, 0.10
GP age	0.01	0.01	0.419	-0.01, 0.03
No. of daily consultations	0.02	0.02	0.326	-0.02, 0.06
				
**GP knowledge about the workplace**				
Time × group (T0 – T1)	0.75	0.20	<0.001	0.35, 1.15
Time × group (T1 – T2)	-0.22	0.20	0.282	-0.63, 0.19
GP gender	-0.02	0.16	0.881	-0.33, 0.28
GP age	0.02	0.01	0.010	0.01, 0.04
No. of daily consultations	0.01	0.02	0.659	-0.02, 0.04
				
**GP knowledge about the work tasks**				
Time × group (T0 – T1)	0.39	0.19	0.049	0.02, 0.77
Time × group (T1 – T2)	-0.18	0.20	0.360	-0.57, 0.21
GP gender	0.04	0.14	0.771	-0.24, 0.33
GP age	0.01	0.01	0.091	-0.00, 0.03
No. of daily consultations	0.01	0.01	0.988	-0.03, 0.03
				
**GP knowledge about perceived stressors**				
Time × group (T0 – T1)	0.55	0.17	0.001	0.23, 0.88
Time × group (T1 – T2)	-0.41	0.17	0.018	-0.74, -0.07
GP gender	0.13	0.12	0.279	-0.11, 0.37
GP age	0.01	0.01	0.025	0.00, 0.03
No. of daily consultations	0.00	0.01	0.829	-0.02, 0.03

The outcome measures were analysed by linear mixed models for repeated measurements using T1, the control group and male GP scores as reference values.

Estimates are adjusted for GP age, gender and number of daily consultations.

### GP knowledge of patient perceived stressors

The GPs reported that they were most informed about their patients' perceived physical stressors at work, less informed about mental and even less informed about work organisational stressors at work (Guttman's reproducibility coefficient: 0.95) at T0. For the intervention group at T1, the Guttman's reproducibility coefficient was: 1.00.

### GP and patient evaluations

Data from 130 pairs of evaluation score-sheets (two pairs were not returned and one patient score-sheet had missing items), 130 filled in by intervention GPs and 130 by their respective patients, was available for statistical analyses. Both the GP and the patients evaluated the GP's knowledge about patient work factors as significantly higher immediately after the consultation, adjusted estimates: 0.57 (95% CI: 0.46, 0.69) and 0.48 (95% CI: 0.38, 0.57), respectively for the five-point scale.

## Discussion

### Summary of main findings

The use of a structured method for functional assessment in general practice led to significantly increased GP knowledge and higher self-efficacy towards functional assessments. In addition, the GPs showed increased knowledge about the patients' workplace and perceived stressors. The intervention effects sustained at the second follow-up six months later. The GPs were better informed about their patients' physical than about their mental and work organisational perceived stressors. Both the intervention GPs and their patients reported increased GP knowledge about patient work factors as a result of the consultation.

### GP knowledge, attitude and self-efficacy towards functional assessments

Earlier studies have shown that active interventions can increase knowledge levels [23-27], although there are exceptions [28]. An increase in self-efficacy has also been reported by others [23,25,26,28]. We found no intervention effect on attitudes towards functional assessments, and failure in changing attitude levels has also been reported by others [27,28]. This could be due to regression to the mean, the phenomenon whereby respondents with extreme values will, for purely statistical reasons, probably give less extreme measurements on other occasions. An alternative explanation is that this study sample of volunteers represented a selected group that already was very positive towards functional ability. Thus, it might have been difficult to achieve further positive changes in attitude, which has also been suggested by others [23,27].

The relatively large increases in GP knowledge and self-efficacy mean scores, 0.6–0.9 on a five-point scale, not only represent statistically significant changes, but probably also reflect clinically relevant changes. The highest change in mean score, 0.9, was found for self-efficacy regarding functional assessments. This increase could be attributed to a combination of increased knowledge about functional assessments along with practice and experience in doing such assessments. Since the intervention provided a "tool" for performing functional assessments, this might have increased the GPs' mastering beliefs.

### GP knowledge of patient perceived stressors

An increase in knowledge about the individual patients' workplace and perceived stressors was expected since the functional assessment method requires that the GP collect more information and spend more time on work related issues in the prolonged consultation. The reason for the significant change in mean score for knowledge about perceived stressors in the control group from T1 to T2 is unknown. At the same time, however, there was a non-significant decrease in mean score in the intervention group. These changes in opposite directions may cause the significant estimate for the interaction term in the longitudinal analyses comparing T1 and T2.

The large potential for increasing GP knowledge about the patient perceived work organisational stressors during the functional assessment was not utilized. This could indicate that the GPs feel more competent to handle and address physical, rather than mental and work organisational factors, in their work with persons on long-term sick leave. The structured functional assessment method treats the three as equally important factors, but maybe more focus should have been given to assess work organisational stressors.

### GP and patient evaluations

The GPs reported a slightly higher (p > 0.05) increase than their patients did, in GP knowledge about patient work factors. A previous study has found high agreement between the GP and the patient when assessing work ability [29].

### Implications for future research or clinical practice

Most intervention GPs implemented the method on a small number of patients. Possibly, the effects of the intervention could have been greater if the number of patients was higher. The GPs selected the patients themselves according to the inclusion and exclusion criteria. However, the patient inclusion criteria used in this study, 'having good prospects of a return to work', is not very specific. It often relies on a subjective judgement by the GPs, but in our opinion the present method is not appropriate in cases where the patient applies for permanent social benefits. We believe, from our contact with the GPs, that the patient selection was random, but it cannot be excluded that the GPs chose patients known to display compliance. This might have given the GPs a skewed impression of the intervention's usefulness. However, the patients were representative for long-term sick-listed persons in relation to age and gender.

This method for functional assessment is quite time-consuming compared to a normal GP consultation in busy and ordinary practice, which is estimated to last for 10–20 minutes. The low implementation rate among the GPs indicates that this method is unlikely to be, and should not be, implemented routinely. In our opinion the method should rather be applied selectively with the most relevant application being cases of complex long-term sick leave where the GP recognise the need for a more thorough assessment of the patient. Also, implementation of the method may be initiated by the local social security officer requesting information on functional ability. By providing such information along with suggestions for workplace adjustments, The Function Assessment Report may facilitate an early return to work.

The findings suggest that a one-day workshop, with some phone support, is sufficient to provide the GPs with adequate background information to apply the structured functional assessment method to persons with long-term sick leave. For future work, it would be interesting to have a critical look at what the GPs wrote in the Function Assessment Forms, whether they pointed out patient resources and if they provided suggestions for workplace adjustments to facilitate a quick return to work. Explorations of the patient self-reported functional ability level and work demands in relation to register based sick leave also represent an interesting possibility for future work.

### Strengths and limitations

The randomised design minimizes the effect of biases that we were unable to control for, and low levels of missing item values contributed to good data quality in this study. The second follow-up gave us a possibility to assess the stability of the intervention effect, and the patients' evaluation of the GPs' knowledge level represents a validation of the GPs' own evaluation.

The number of GPs included in the study was low compared to the number of invited GPs, but according to power analyses, the number of GPs in each group was satisfactory. Like in other studies [23,28], it proved difficult to recruit GPs on a voluntary basis for a clinical study.

The sample is representative for general practice with regard to age and gender. At the same time they probably belong to a highly selected group that is more interested in functional assessments than many other GPs are. Such self-selection bias was unavoidable and probably reflects that mainly interested GPs voluntarily seek special skills and utilise structured methods in this domain.

The five intervention GPs that withdrew after the randomisation raises the possibility of post-randomisation selection bias, thus representing a study weakness. As we have no collected data for these persons, we cannot do drop-out analysis for these five GPs. Further, it means that no true intention-to-treat principle can be followed in this study.

The use of self-reporting rather than objective measures for the study outcomes represents another limitation in this study. Along with the lack of blinding of the GPs, it might have led to bias for the positive results in this study. However, it represented a feasible way of measuring different components of the intervention, and objective measures of register based patient sick leave will be reported elsewhere.

## Conclusion

This study showed that a structured functional assessment method enhanced the GPs' knowledge about functional assessments and patient work factors, as well as their self-efficacy towards performing functional assessments. A one-day workshop and phone support provided the GPs with adequate background information to apply these assessments to persons with long-term sick leave. The intervention effects sustained at the follow-up six months later.

## Competing interests

The authors declare that they have no competing interests.

## Authors' contributions

NØ planned and designed the study, collected the data, performed most statistical analyses, drafted the manuscript and coordinated the study. PG participated in the planning and designing of the study, interpretation of the results and in drafting the manuscript. JSB assisted the statistical analyses and reviewed the manuscript. DH performed the factor analysis, the Guttman's reproducibility coefficient analysis, assisted other statistical analyses and reviewed the manuscript. SB planned and designed the study, participated in the interpretation of results and in drafting and revising the manuscript. All authors have read and approved the final manuscript.

## Pre-publication history

The pre-publication history for this paper can be accessed here:



## Supplementary Material

Additional file 1 **The Norwegian Function Assessment Form.** Click here for file

Additional file 2 **The Work Description Form.** Click here for file

Additional file 3 **The Function Assessment Report.** Click here for file

Additional file 4 **Main Questionnaire.** Click here for file

Additional file 5 **GP Evaluation Score-sheet.** Click here for file

Additional file 6 **Patient Evaluation Score-sheet.** Click here for file

## References

[B1] Organisation for Economic Co-operation and Development (OECD) (2003). Transforming Disability into Ability Policies to Promote Work and Income Security for Disabled People.

[B2] Brage S, Donceel P, Falez F (2008). Development of ICF core set for disability evaluation in social security. Disabil Rehabil.

[B3] Krohne K, Brage S (2007). New rules meet established sickness certification practice. A focus group study on the introduction of functional assessments in Norwegian primary care. Scand J Prim Health Care.

[B4] Andersen EJ (2003). [Functional assessments causing trouble? (in Norwegian]. Tidsskr Nor Laegeforen.

[B5] Swales K, Craig P (1997). Evaluation of the Incapacity Benefit Medical Test: In-house report 26.

[B6] Tuomi K, Ilmarinen J, Jahkola A, Katajarinne L, Tulkki A (1998). Work Ability Index.

[B7] Nygard CH, Eskelinen L, Suvanto S, Tuomi K, Ilmarinen J (1991). Associations between functional capacity and work ability among elderly municipal employees. Scand J Work Environ Health.

[B8] Grimshaw JM, Shirran L, Thomas R, Mowatt G, Fraser C, Bero L (2001). Changing provider behavior: an overview of systematic reviews of interventions. Med Care.

[B9] Altman DG (1991). Practical statistics for medical research.

[B10] Brage S, Fleten N, Knudsrod OG, Reiso H, Ryen A (2004). [Norwegian Functional Scale – a new instrument in sickness certification and disability assessments (in Norwegian)]. Tidsskr Nor Laegeforen.

[B11] World Health Organization (2001). ICF-International Classification of Functioning, Disability, and Health.

[B12] Osteras N, Brage S, Garratt A, Benth JS, Natvig B, Gulbrandsen P (2007). Functional ability in a population: normative survey data and reliability for the ICF based Norwegian Function Assessment Scale. BMC Public Health.

[B13] Osteras N, Gulbrandsen P, Garratt A, Benth JS, Dahl FA, Natvig B (2008). A randomised comparison of a four- and a five-point scale version of the Norwegian Function Assessment Scale. Health Qual Life Outcomes.

[B14] Rogers EM (1995). Diffusion of innovation.

[B15] Bandura A (1977). Self-efficacy: toward a unifying theory of behavioral change. Psychol Rev.

[B16] Arbuckle J, Wothke W (1999). Amos 40 User's guide.

[B17] Akaike H (1974). A new look at the statistical model identification. IEEE Transactions on Automatic Control.

[B18] Fitzmaurice GM, Laird NM, Ware JH (2004). Applied longitudinal analysis.

[B19] Massof RW (2004). Likert and Guttman scaling of visual function rating scale questionnaires. Ophthalmic Epidemiol.

[B20] Hays RD, Ellickson PL (1990). Guttman scale analysis of longitudinal data: a methodology and drug use applications. Int J Addict.

[B21] Norwegian Insurance Administration (2003). [Yearbook of National Insurance Statistics 2003 (in Norwegian)].

[B22] Norwegian Labour and Welfare Administration [Principal number report, per 4 quarter 2005 (in Norwegian] [Online] 10-2-2006 [cited 21-12-2006].

[B23] Sanci LA, Coffey CM, Veit FC, Carr-Gregg M, Patton GC, Day N (2000). Evaluation of the effectiveness of an educational intervention for general practitioners in adolescent health care: randomised controlled trial. BMJ.

[B24] Forsetlund L, Bradley P, Forsen L, Nordheim L, Jamtvedt G, Bjorndal A (2003). Randomised controlled trial of a theoretically grounded tailored intervention to diffuse evidence-based public health practice [ISRCTN23257060]. BMC Med Educ.

[B25] Margalit AP, Glick SM, Benbassat J, Cohen A, Katz M (2005). Promoting a biopsychosocial orientation in family practice: effect of two teaching programs on the knowledge and attitudes of practising primary care physicians. Med Teach.

[B26] Short LM, Surprenant ZJ, Harris JM (2006). A community-based trial of an online intimate partner violence CME program. Am J Prev Med.

[B27] Shuval K, Berkovits E, Netzer D, Hekselman I, Linn S, Brezis M (2007). Evaluating the impact of an evidence-based medicine educational intervention on primary care doctors' attitudes, knowledge and clinical behaviour: a controlled trial and before and after study. J Eval Clin Pract.

[B28] King M, Davidson O, Taylor F, Haines A, Sharp D, Turner R (2002). Effectiveness of teaching general practitioners skills in brief cognitive behaviour therapy to treat patients with depression: randomised controlled trial. BMJ.

[B29] Reiso H, Nygard JF, Brage S, Gulbrandsen P, Tellnes G (2000). Work ability assessed by patients and their GPs in new episodes of sickness certification. Fam Pract.

