# Supplementary material for: Implementing structured functional assessments in general practice for persons with long-term sick leave: a cluster randomised controlled trial
Source: BMC Fam Pract. 2009 May 6;10:31. doi: 10.1186/1471-2296-10-31 (PMC2688495; doi:10.1186/1471-2296-10-31)
Supplement: Additional file 1 — The Norwegian Function Assessment Form. [file 1471-2296-10-31-S1.pdf]

Norwegian Function Assessment Scale

When you have filled in the form, please bring it with you to your doctor

During the last week, have you had difficulty doing the following activities because of your health? Please put a cross in the box for the best answer for each question. Even if a question does not seem to fit your circumstances, please try to answer it as best you can. If a question is not relevant for you, for example because you do not drive a car, you can draw a line through the question.

**Have you had difficulty doing the following activities during the last week:**	No difficulty	Mild difficulty	Moderate difficulty	Much difficulty	Could not do it
**Walking/standing**					
Standing	☐	☐	☐	☐	☐
Walking less than a kilometre on flat ground	☐	☐	☐	☐	☐
Walking more than a kilometre on flat ground	☐	☐	☐	☐	☐
Walking on different surfaces	☐	☐	☐	☐	☐
Going up and down stairs	☐	☐	☐	☐	☐
Going shopping for your groceries	☐	☐	☐	☐	☐
Putting on your shoes and socks	☐	☐	☐	☐	☐
**Holding /picking up things**					
Picking up a coin from a table with your fingers	☐	☐	☐	☐	☐
Holding and turning a steering wheel	☐	☐	☐	☐	☐
Driving a car	☐	☐	☐	☐	☐
Preparing food	☐	☐	☐	☐	☐
Writing	☐	☐	☐	☐	☐
Performing everyday tasks on your own	☐	☐	☐	☐	☐
Engaging in your leisure activities	☐	☐	☐	☐	☐
Putting on and taking off your clothes	☐	☐	☐	☐	☐
**Lifting/carrying**					
Lifting an empty soda bottle crate from the floor	☐	☐	☐	☐	☐
Carrying shopping bags in your hands	☐	☐	☐	☐	☐
Carrying a little sack/backpack on your shoulders or back	☐	☐	☐	☐	☐
Pushing and pulling with your arms	☐	☐	☐	☐	☐
Cleaning your house	☐	☐	☐	☐	☐
Washing your clothes	☐	☐	☐	☐	☐

Norwegian Function Assessment Scale

When you have filled in the form, please bring it with you to your doctor.

**Have you had difficulty doing the following activities during the last week:**	No difficulty	Mild difficulty	Moderate difficulty	Much difficulty	Could not do it
**Sitting**					
Sitting on a kitchen chair	☐	☐	☐	☐	☐
Riding as a passenger in a car	☐	☐	☐	☐	☐
Riding as a passenger on public transport	☐	☐	☐	☐	☐
**Managing**					
Staying alert and being able to concentrate	☐	☐	☐	☐	☐
Working in groups	☐	☐	☐	☐	☐
Guiding others in their activities	☐	☐	☐	☐	☐
Managing everyday responsibility	☐	☐	☐	☐	☐
Managing everyday stress and strains	☐	☐	☐	☐	☐
Managing to take criticism	☐	☐	☐	☐	☐
Managing to control your anger and aggression	☐	☐	☐	☐	☐
**Cooperation/communication**					
Remembering things	☐	☐	☐	☐	☐
Understanding spoken messages	☐	☐	☐	☐	☐
Understanding written messages	☐	☐	☐	☐	☐
Speaking	☐	☐	☐	☐	☐
Participating in a conversation with many people	☐	☐	☐	☐	☐
Using the telephone	☐	☐	☐	☐	☐
**Senses**					
Watching television	☐	☐	☐	☐	☐
Listening to the radio	☐	☐	☐	☐	☐
