# Supplementary material for: Implementing structured functional assessments in general practice for persons with long-term sick leave: a cluster randomised controlled trial
Source: BMC Fam Pract. 2009 May 6;10:31. doi: 10.1186/1471-2296-10-31 (PMC2688495; doi:10.1186/1471-2296-10-31)
Supplement: Additional file 2 — The Work Description Form. [file 1471-2296-10-31-S2.pdf]

Work Description Form

When you have filled in the form, please bring it with you to the doctor

Type of work: ……………………………………………………………………………………

For how long have you been employed? ☐ Less than a year ☐ 1-5 years ☐ More than 5 years

Employment status: ☐ In work/employed ☐ Unemployed ☐ Rehabilitation

Do you work full- or part-time? ☐ Full-time ☐ Part-time

Name three positive aspects of your work:

1_____________________ 2_____________________ 3____________________

Do you feel your work is physically straining? ☐ No ☐ Yes

If YES, tick off appropriate box(es)

- ☐ Much sitting
- ☐ Standing still
- ☐ Much walking
- ☐ Kneeling or squatting
- ☐ Working with arms lifted/reached forward
- ☐ Lifting many heavy loads
- ☐ Doing precise movements with hands
- ☐ Doing the same movements many times a minute
- ☐ Working on/with vibrating surface/tools
- ☐ Must hold the same position for long periods
- ☐ Heavy work
- ☐ Other:……………….

Do you feel your work is mentally straining? ☐ No ☐ Yes

If YES, tick off appropriate box(es)

- ☐ Have to be alert and concentrated
- ☐ Have to deal with emotions
- ☐ Have to have good memory
- ☐ Other:…………………..
- ☐ Have to be creative
- ☐ Working with colleagues on tasks
- ☐ Direct client/customer/student contact

Do you feel that the work organization is straining? ☐ No ☐ Yes

If YES, tick off appropriate box(es)

- ☐ Have shift work
- ☐ Working by contract
- ☐ Have work with high season intensity
- ☐ Have management responsibilities
- ☐ Have too much to do
- ☐ Have too much responsibility
- ☐ Other:………………….
- ☐ Unclear what is expected at work
- ☐ Cannot set work pace myself
- ☐ Cannot decide myself when to take breaks
- ☐ Do not get help with the heaviest tasks
- ☐ Get little support and help from superiors
- ☐ Do not feel that my work effort is appreciated
