# Supplementary material for: Implementing structured functional assessments in general practice for persons with long-term sick leave: a cluster randomised controlled trial
Source: BMC Fam Pract. 2009 May 6;10:31. doi: 10.1186/1471-2296-10-31 (PMC2688495; doi:10.1186/1471-2296-10-31)
Supplement: Additional file 3 — The Function Assessment Report. [file 1471-2296-10-31-S3.pdf]

Function Assessment Report

Medical functional assessment filled out on request from the National Insurance Scheme or employer.

This form is to be filled out by treating physician in consultation with the patient, and to be seen in light of the Norwegian Function Assessment Scale, the Work Description Form and Key Questions.

Patient name………………………………………………………… Date of birth ☐☐☐☐☐☐

Weeks on sick leave ☐☐ weeks

Physician name:……………………………………………… Date of consultation ☐☐☐☐☐☐

1. Functional ability (from Norwegian Function Assessment Scale)

Check the box indicating functional resources or limitations as stated by the patient.

FUNCTION DOMAIN	Resources	Limitations
**Walking/standing**		
**Holding/picking up things**		
**Lifting/carrying**		
**Sitting**		
**Managing**		
**Cooperation/communication**		
**Senses**		

Comments:

Work ability (from Norwegian Function Assessment Scale)

Check the box indicating degree of reduced work ability as stated by the patient

Hardly reduced at all	Not much reduced	Moderately reduced	Much reduced	Very much reduced
☐	☐	☐	☐	☐

2. Work description (from Work Description Form)

Is the patient's work straining? Check the box and explain in the column to the right what is perceived as straining.

Work description	No	Yes	If yes, what is perceived as straining?
Physically straining			
Mentally straining			
Straining due to work organization			

TURN PAGE

3. Physician assessment of functional resources and limitations in relation to patient's work tasks

Which are the patient's resources that can be used as means in returning to work?

Does the patient have special needs, *e.g.* for breaks and rest?

Can the patient work part-time? ☐ yes ☐ no

4. Medical treatment

Will ongoing or planned medical treatment influence the patient's functional ability? Will treatment interfere with activity? When will treatment be finished?

5. Protective needs

State situations or external conditions that the patient should avoid for medical reasons, *eg*. lifting/carrying, working with elevated arms, or contact with costumers, students or clients.

6. Physician suggestions for workplace measures

Will adjustments or supportive aids facilitate the patient's return to work?.

☐ Probably yes ☐ Do not know ☐ Probably no

Concrete suggestions for adjustments at place of work. What can facilitate return to work?

7. Comments

....	....	……………………………….
Date	Physician's signature	Patient's signature
