# Supplementary material for: Implementing structured functional assessments in general practice for persons with long-term sick leave: a cluster randomised controlled trial
Source: BMC Fam Pract. 2009 May 6;10:31. doi: 10.1186/1471-2296-10-31 (PMC2688495; doi:10.1186/1471-2296-10-31)
Supplement: Additional file 4 — Main Questionnaire. [file 1471-2296-10-31-S4.pdf]

UNIVERSITY OF OSLO

MAIN QUESTIONNAIRE

By "Work related activities" we mean here: part-time sick leave, active sick leave, vocational rehabilitation, lump sum grant for workplace adjustments or grant for travel expenses. Long-term sick leave is defined as GP certified sick leave lasting for more than eight weeks.

Tick off one box for each line.

**How do you rate your knowledge about:**	**Very poor**	**Poor**	**Medium**	**Good**	**Very good**
1. Functional assessments for patients with long-term sick leave	☐	☐	☐	☐	☐
2. Vocational rehabilitation for patients with long-term sick leave	☐	☐	☐	☐	☐
3. Work related activities for patients with long-term sick leave	☐	☐	☐	☐	☐

**What is your opinion on the following statements?**	**Totally disagree**	**Partly disagree**	**Neither agree nor disagree**	**Partly agree**	**Totally agree**
4. Functional assessments are important for reintegrating the long-term sick-listed persons into working life?	☐	☐	☐	☐	☐
5. Vocational rehabilitation is important for reintegrating the long-term sick-listed persons into working life?	☐	☐	☐	☐	☐
6. Work related activities are important for reintegrating the long-term sick-listed persons into working life?	☐	☐	☐	☐	☐
7. Functional assessments are important for the social security officers in aiding the long-term sick-listed persons to reintegrate into working life?	☐	☐	☐	☐	☐
8. Functional assessments are important for the employer for knowing how to adjust the work/ workplace for the long-term sick-listed persons?	☐	☐	☐	☐	☐
9. Performing functional assessments on long-term sick-listed patiens is meaningful?	☐	☐	☐	☐	☐

Turn the page

**What is your opinion on the following statements:**	**Totally disagree**	**Partly disagree**	**Neither agree nor disagree**	**Partly agree**	**Totally agree**
10. Performing functional assessments on long-term sick listed patiens is a waste of time	☐	☐	☐	☐	☐
11. I consider my self well skilled for performing functional assessments on my long-term sick- listed patients	☐	☐	☐	☐	☐
12. Functional assessments on patients with long-term sick leave should be performed by other occupational groups than general practitioners	☐	☐	☐	☐	☐
13. I am convinced that I can perform a functional assessment on a long-term sick-listed patient if his or her employer or the local social security officer request that	☐	☐	☐	☐	☐
14. I am convinced that I know how I can assess work relevant resources on a long-term sick-listed patient	☐	☐	☐	☐	☐

**How do you rate your knowledge about:**	**Very poor**	**Poor**	**Medium**	**Good**	**Very good**
15. The workplaces for your long-term sick-listed patients?	☐	☐	☐	☐	☐
16. The worktasks for your long-term sick-listed patients?	☐	☐	☐	☐	☐
17. Your long-term sick-listed patients' perceived physical stressors at work?	☐	☐	☐	☐	☐
18. Your long-term sick-listed patients' perceived mental stressors at work?	☐	☐	☐	☐	☐
19. Your long-term sick-listed patients' perceived work organisational stressors at work?	☐	☐	☐	☐	☐
