# Supplementary material for: Implementing structured functional assessments in general practice for persons with long-term sick leave: a cluster randomised controlled trial
Source: BMC Fam Pract. 2009 May 6;10:31. doi: 10.1186/1471-2296-10-31 (PMC2688495; doi:10.1186/1471-2296-10-31)
Supplement: Additional file 5 — GP Evaluation Score-sheet. [file 1471-2296-10-31-S5.pdf]

UNIVERSITY OF OSLO

QUESTIONS FOR **THE GP** AFTER THE CONSULTATION

Consultation date: ☐☐☐☐☐☐

Patient gender: Male ☐ Female ☐

Patient year of birth: ☐☐☐☐

**How do you rate your knowledge about:**	**No knowledge**	**Some**	**Good**	**Very good**	**Exceptionally good**
1. this patient's workplace, work tasks and work demands <u>before</u> today's consultation?	☐ 1	☐ 2	☐ 3	☐ 4	☐ 5
2. this patient's workplace, work tasks and work demands <u>after</u> today's consultation?	☐ 1	☐ 2	☐ 3	☐ 4	☐ 5
