# Supplementary material for: Implementing structured functional assessments in general practice for persons with long-term sick leave: a cluster randomised controlled trial
Source: BMC Fam Pract. 2009 May 6;10:31. doi: 10.1186/1471-2296-10-31 (PMC2688495; doi:10.1186/1471-2296-10-31)
Supplement: Additional file 6 — Patient Evaluation Score-sheet. [file 1471-2296-10-31-S6.pdf]

UNIVERSITY OF OSLO

QUESTIONS FOR **THE PATIENT** AFTER THE CONSULTATION

Consultation date: ☐☐☐☐☐☐

Gender: Male ☐ Female ☐

Year of birth: ☐☐☐☐

**How do you rate your GP's knowledge about:**	**No knowledge**	**Some**	**Good**	**Very good**	**Exceptionally good**
1. your workplace, work tasks and work demands <u>before</u> today's consultation?	☐1	☐2	☐3	☐4	☐5
2. your workplace, work tasks and work demands <u>after</u> today's consultation?	☐1	☐2	☐3	☐4	☐5
